# Supplementary material for: Assessing soil compaction and micro-topography impacts of alternative heather cutting as compared to burning as part of grouse moor management on blanket bog
Source: PeerJ. 2019 Jul 18;7:e7298. doi: 10.7717/peerj.7298 (PMC6642791; doi:10.7717/peerj.7298)
Supplement: Supplemental Information 4 — This document provides Google Earth pictures as Figures S1 (Google Earth ©2019), S2, and S3 for each study site (Nidderdale, Mossdale and Whitendale, respectively) together with their catchment outlines (red: burnt & green: mown) and indications of the 5 × 5 m plot locations (indicated as squares) and the automated weather station location (AWS). Note that all pictures show the sites before management started (which was in 2013). [file peerj-07-7298-s004.docx]

This document provides Google Earth pictures as supplementary **Figures (1-3)** for each study site (Nidderdale, Mossdale and Whitendale, respectively) together with their catchment outlines (red: burnt & green: mown) and indications of the 5 x 5 m plot locations (indicated as squares) and the automated weather station location (AWS). Note that all pictures show the sites before management started (which was in 2013). The bright green and orange outlines represent the individual management areas (mown and burnt, respectively) with plots contained within them.


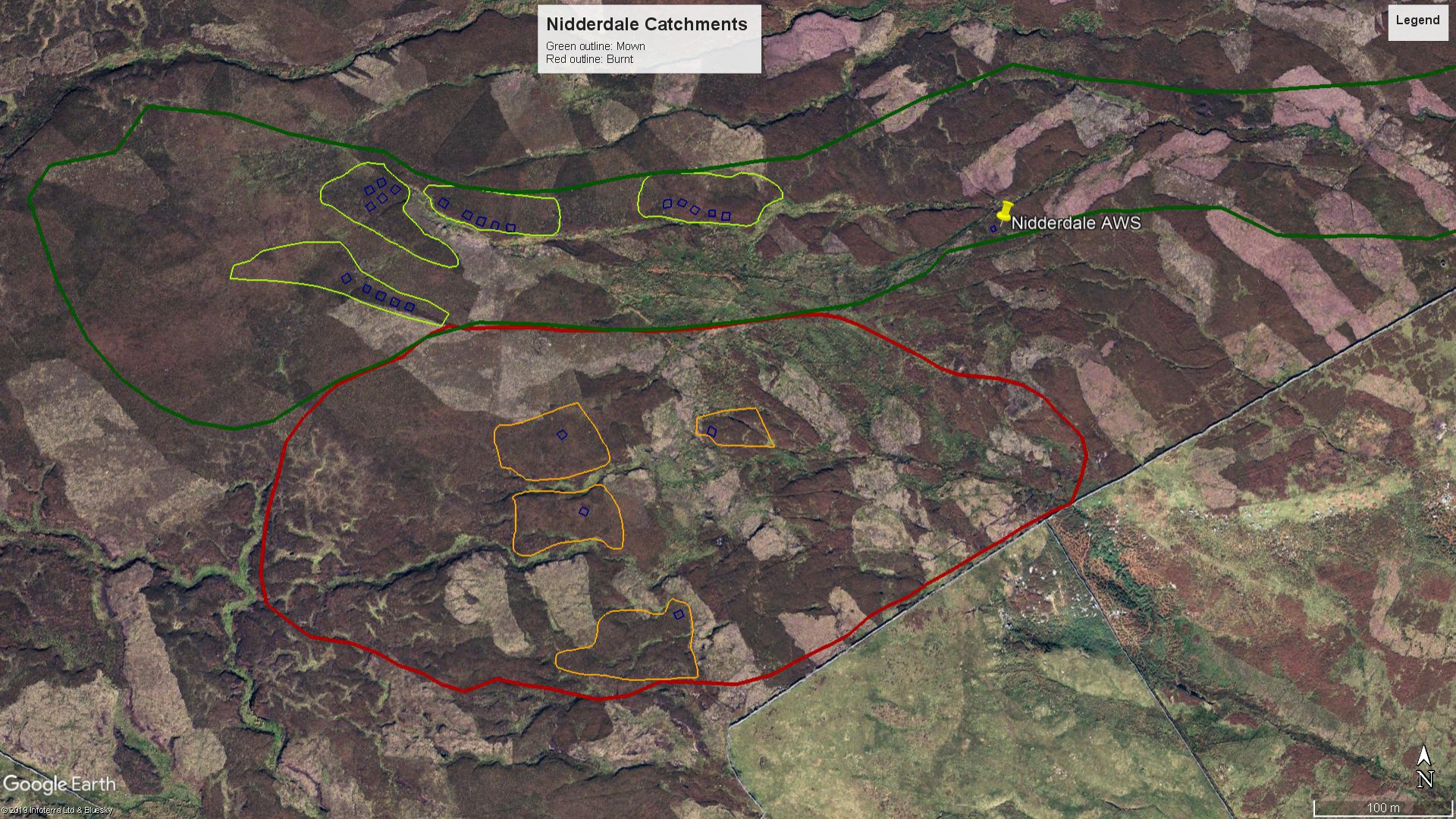


**Figure 1_S** Google Earth picture (2009) for the Nidderdale site.


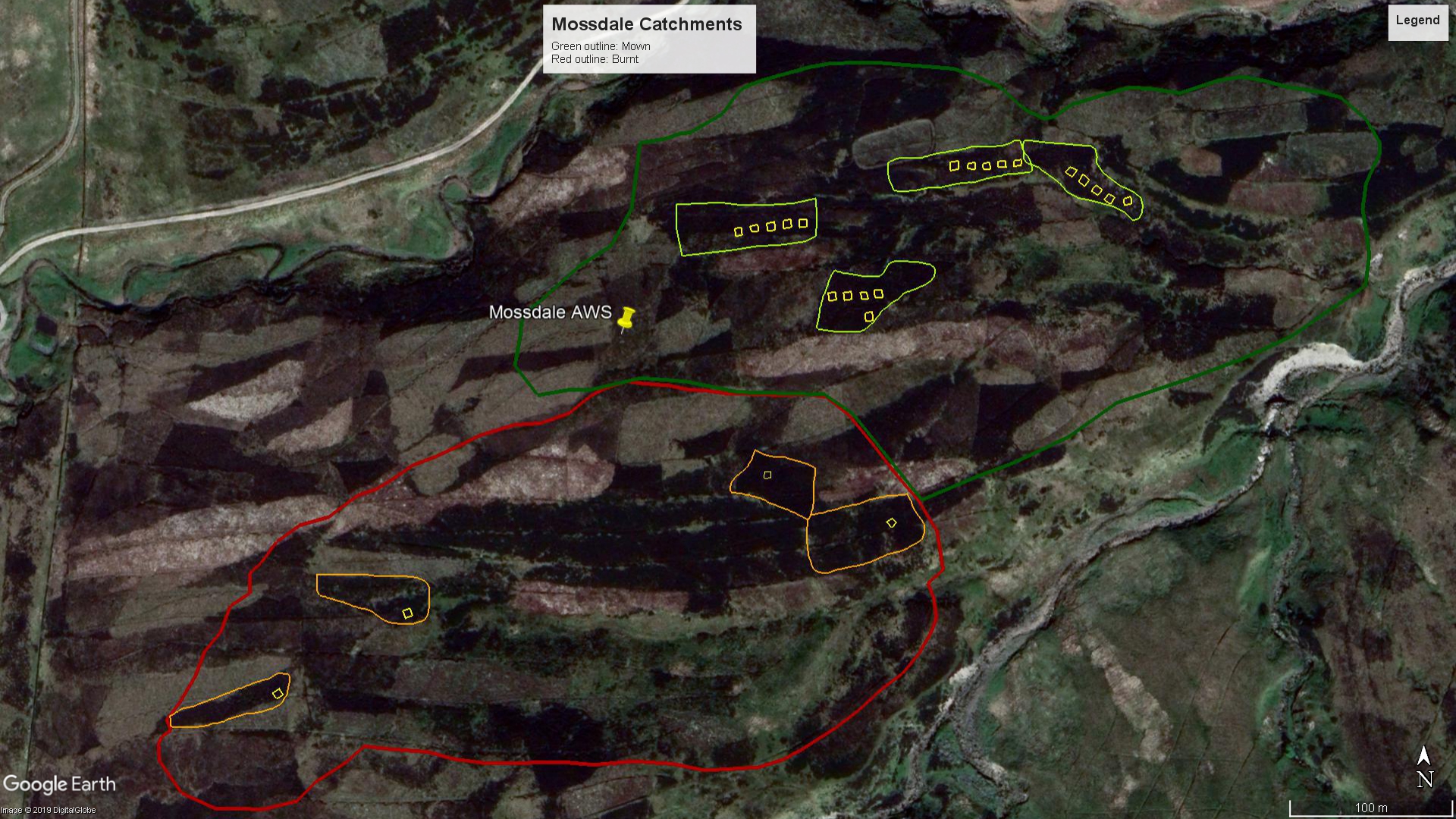


**Figure 2_S** Google Earth picture (2011) for the Mossdale site.


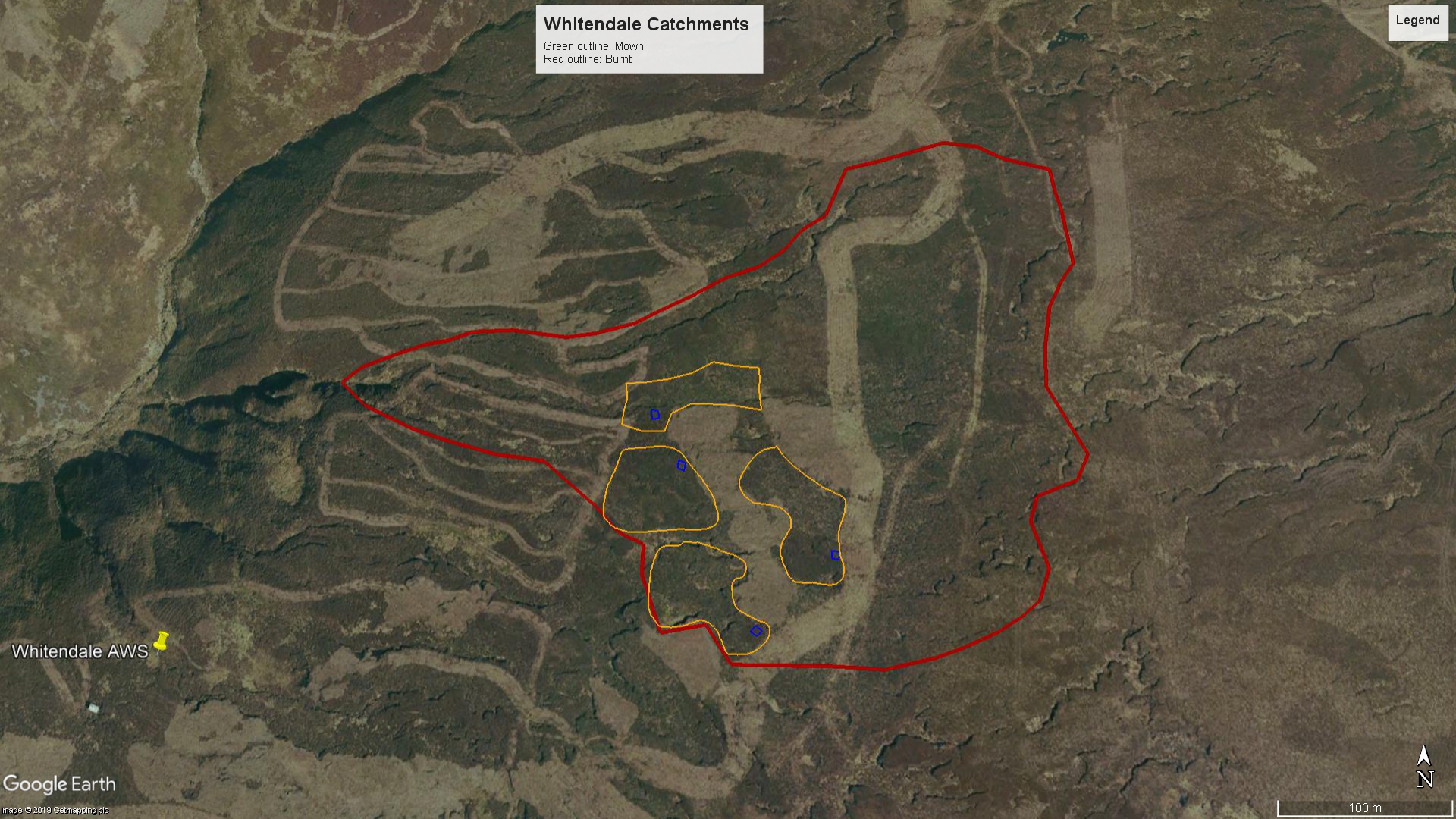


**Figure 3a_S** Google Earth picture (2010) for the Whitendale burnt sub-catchment site. Shown are also previous mown areas (2007 & 2009).


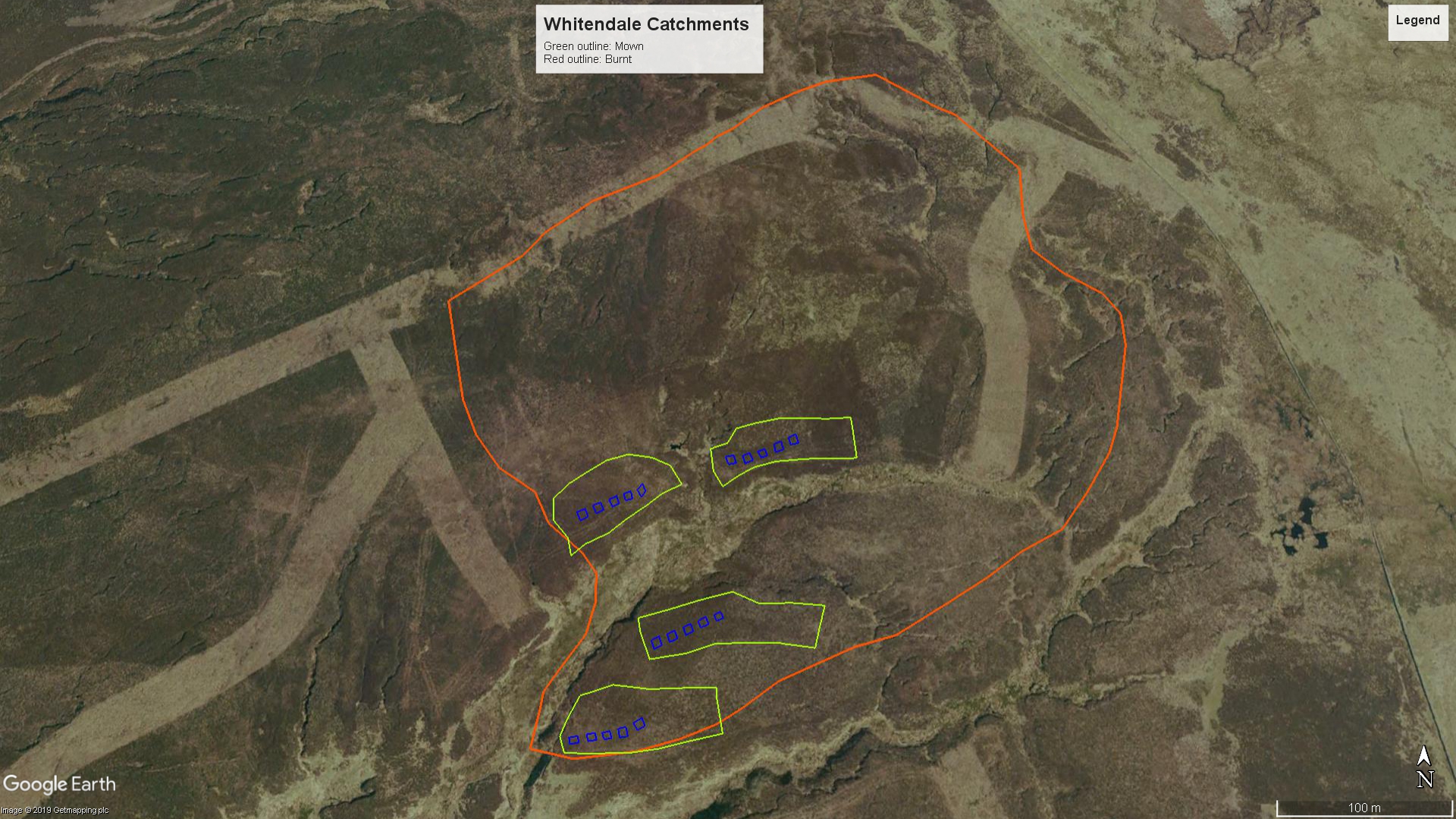


**Figure 3b_S** Google Earth picture (2010) for the Whitendale mown sub-catchment site. Shown are also previous mown areas (2007 & 2009).
